# Investigating the Effect and Mechanism of 3-Methyladenine Against Diabetic Encephalopathy by Network Pharmacology, Molecular Docking, and Experimental Validation

**DOI:** 10.3390/ph18050605

**Published:** 2025-04-22

**Authors:** Jiaxin Chu, Jianqiang Song, Zhuolin Fan, Ruijun Zhang, Qiwei Wang, Kexin Yi, Quan Gong, Benju Liu

**Affiliations:** Department of Medcine, Yangtze University, Jingzhou 434023, China; chujiaxin@aliyun.com (J.C.);

**Keywords:** 3-methyladenine, diabetic encephalopathy, network pharmacology, molecular docking, AKT/GSK-3β, mechanism

## Abstract

**Background/Objectives:** Diabetic encephalopathy (DE), a severe neurological complication of diabetes mellitus (DM), is characterized by cognitive dysfunction. 3-Methyladenine (3-MA), a methylated adenine derivative, acts as a biomarker for DNA methylation and exhibits hypoglycemic and neuroprotective properties. However, the pharmacological mechanisms underlying 3-MA’s therapeutic effects on diabetic microvascular complications remain incompletely understood, owing to the intricate and multifactorial pathogenesis of DE. **Methods**: This study employed network pharmacology and molecular docking techniques to predict potential targets and signaling pathways of 3-MA against DE, with subsequent validation through animal experiments to elucidate the molecular mechanisms of 3-MA in DE treatment. **Results**: Network pharmacological analysis identified two key targets of 3-MA in DE modulation: AKT and GSK3β. Molecular docking confirmed a strong binding affinity between 3-MA and AKT/GSK3β. In animal experiments, 3-MA significantly reduced blood glucose levels in diabetic mice, ameliorated learning and memory deficits, and preserved hippocampal neuronal integrity. Furthermore, we found that 3-MA inhibited apoptosis by regulating the expression of Bax and BCL-2. Notably, 3-MA also downregulated the expression of amyloid precursor protein (APP) and Tau while enhancing the expression of phosphorylated AKT and GSK-3β. **Conclusions**: Our findings may contribute to elucidating the therapeutic mechanisms of 3-MA in diabetic microangiopathy and provide potential therapeutic targets through activation of the AKT/GSK-3β pathway.

## 1. Introduction

With the global incidence of diabetes continuing to rise and the increasing life expectancy of the population, the prevalence of diabetic encephalopathy (DE) has significantly increased, posing a substantial public health burden [1]. It is projected that the number of individuals with diabetes worldwide will exceed 640 million by 2040. Research indicates that the prevalence of diabetes-related cognitive impairment has reached 13.5%, with even higher rates observed among elderly diabetic patients. Specifically, the prevalence in patients aged 75 years and older may surpass 24.2% [2].

Diabetes mellitus (DM) is a prevalent and serious chronic metabolic disorder that can result in numerous complications. Among these, DE, also referred to as diabetic cognitive impairment, represents one of the most detrimental chronic microvascular complications of DM affecting the central nervous system [3]. DE is characterized by cognitive decline, memory deficits, and morphological and structural alterations in the hippocampal tissues of the brain, which may progress to dementia in severe cases. Both type 1 and type 2 diabetes patients are at risk of developing neuropathy and neurobehavioral abnormalities, significantly impairing their quality of life and overall prognosis.

Tau protein is a potential biomarker for the progression of DE. Overexpression of amyloid precursor protein (APP) promotes Tau phosphorylation, which subsequently induces the formation of neurofibrillary tangles [4,5]. Aberrant phosphorylation of Tau protein is indicative of axonal damage in neurons. Studies have demonstrated that the development of insulin resistance or hyperinsulinemia in diabetic patients elevates both intracellular and extracellular β-amyloid (Aβ) levels, activates insulin signaling through the GSK-3β cascade, and ultimately leads to Aβ accumulation and abnormal Tau phosphorylation [6]. Furthermore, relevant research has shown that pathological manifestations, such as hyperphosphorylation of Tau proteins, are observed in the brains of diabetic patients, accompanied by symptoms of cognitive dysfunction. These manifestations not only adversely affect daily life but may also signify the onset of Alzheimer’s disease [7].

The currently recognized pathogenesis of DE is closely associated with hyperglycemic toxicity [8], vascular endothelial cell injury [9], neuroinflammation [10], neuronal apoptosis [11], insulin resistance [12], Aβ deposition, and Tau protein hyperphosphorylation. These pathological changes ultimately result in cognitive decline, neuronal damage, and neurological complications. However, despite extensive research into the pathomechanisms of DE over many years, the identification of precise drug targets remains in its early stages. Current pharmacological and non-pharmacological treatments can slow disease progression and improve quality of life but do not offer a complete cure [13]. The primary objectives of medication include controlling blood glucose levels, reducing blood pressure and blood lipids, and minimizing the risk of cerebrovascular complications. Commonly used drugs include insulin, metformin, sulfonylureas, sodium–glucose cotransporter 2 (SGLT2) inhibitors, dipeptidyl peptidase 4 (DPP-4) inhibitors, and statins [14,15]. However, these medications may induce adverse effects such as hypoglycemia, hypotension, gastrointestinal disturbances (e.g., diarrhea, nausea, and vomiting), and other complications. Additionally, they may lead to ketoacidosis (particularly in patients with type 1 diabetes), abnormal liver function, and, in more severe cases, infections, fractures, lactic acidosis, and even cancer [16,17]. Consequently, there is an urgent need to explore alternative therapeutic options with improved efficacy and safety profiles.

3-Methyladenine (3-MA) is a methylated derivative of adenine, commonly utilized as an in vivo marker of DNA methylation. It primarily functions as an autophagy inhibitor by targeting phosphatidylinositol 3-kinase catalytic subunit type 3 (PIK3C3/Vps34) to suppress autophagy [18]. Autophagy is a conserved metabolic pathway that plays a critical role in eukaryotic cells [19]. It maintains intracellular homeostasis by degrading metabolic waste through the lysosomal pathway. Positive regulation of autophagy can effectively clear abnormally aggregated proteins in the nervous system, such as Aβ and Tau proteins [20], thereby delaying disease progression. Conversely, excessive activation of autophagy may exacerbate neuronal apoptosis [21]. Studies have demonstrated that 3-MA exhibits blood glucose-lowering effects [22], protects against oxidative stress [23], and inhibits cellular apoptosis [24]. Additionally, 3-MA has shown numerous other beneficial effects, expanding its potential applications beyond diabetic encephalopathy [25]. Growing evidence suggests that 3-MA possesses neuroprotective properties. For instance, in a mouse model of Alzheimer’s disease combined with chronic cerebral hypoperfusion (CCH), 3-MA was found to reverse cognitive decline, reduce Aβ deposition, and mitigate mitochondrial deficits in AD + CCH mice [26].

Given the demonstrated positive effects of 3-MA on reducing blood glucose levels and mitigating neurodegeneration in animal models, we hypothesized that it could serve as an effective therapeutic agent for DE. Therefore, the aim of this study was to investigate the therapeutic effects and underlying mechanisms of 3-MA on cognitive impairment in streptozotocin (STZ)-induced diabetic mice, thereby providing a theoretical foundation for the potential clinical application of 3-MA in the treatment of diabetic cognitive impairment.

## 2. Results

### 2.1. Collection of 3-MA Targets and DE Targets

**Figure 1:**
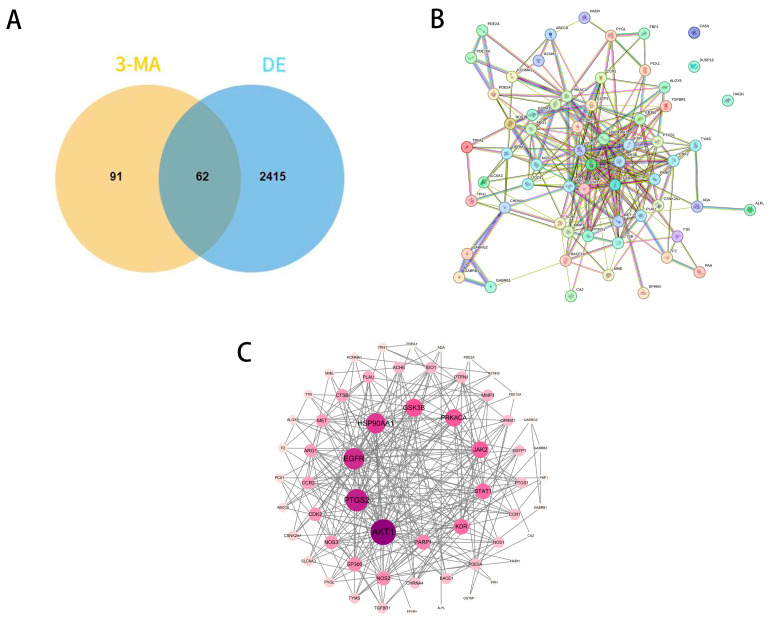
PPI network of common targets of 3-MA and DE. (**A**) Venn diagram of 3-MA target genes and DE-related genes. (**B**) PPI network for the targets of the intersection of 3-MA and DE. (**C**) Potential target PPI network diagram.

A total of 153 potential targets of 3-MA were identified through screening using the Swiss Target Prediction database, the PharmMapper database, and the Similarity Ensemble Approach (SEA) database. Additionally, 3884, 642, and 21 DE-related targets were retrieved from the GeneCards, OMIM, and DisGeNET databases, respectively. After removing duplicate entries, a final set of 2477 DE-related targets was obtained. Among these, 62 targets were found to overlap between 3-MA and DE, as illustrated in the Venn diagram below (Figure 1A).

### 2.2. PPI Network Analysis and Core Target Screening

This initial network analysis was validated using the STRING database for protein–protein interactions in Homo sapiens (confidence ≥ 0.4), and the PPI network map (Figure 1B) was drawn. Then, STRING data were imported into Cytoscape version 3.10.0, and the PPI interaction network (Figure 1C) was drawn in detail (with 59 actual PPI network targets due to the existence of 3 free targets). The data show that 59 nodes represent target proteins, 265 edges represent the interrelationships between target proteins, and the darker nodes indicate higher degree values, indicating a strong correlation between these targets and DE. Topological analysis was performed with the plug-in tool “cyto NCA”, where the top ten targets (degree > 14) were AKT 1, PTGS 2, EGFR, HSP90AA 1, GSK 3β, PRKACA, JAK 2, STAT 1, KDR, and PARP 1.

### 2.3. GO and KEGG Enrichment Analysis

**Figure 2:**
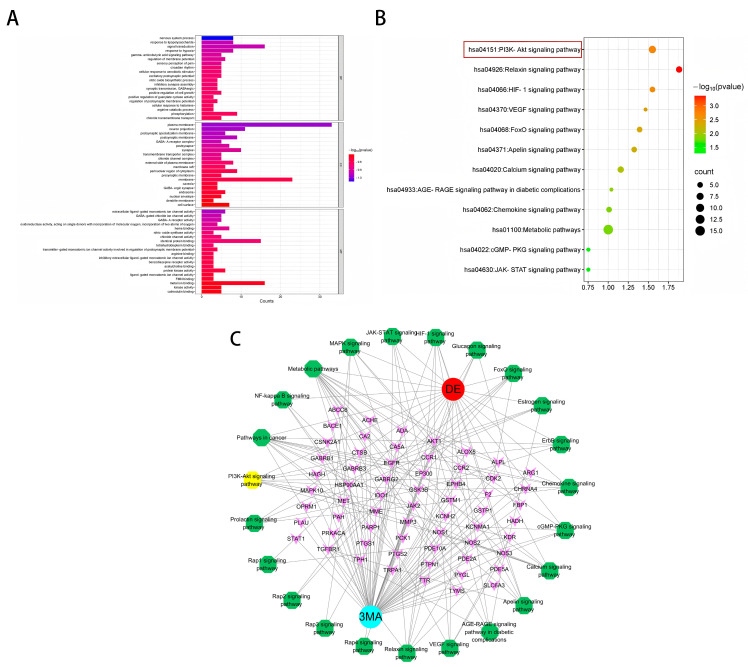
(**A**) GO functional annotation. (**B**) KEGG pathway enrichment analysis. (**C**) Target–pathway network diagram for 3-MA in the treatment of DE. Blue for drugs, red for diseases, yellow and green for pathways, and pink for targets.

A comprehensive Gene Ontology (GO) analysis identified a total of 177 entries associated with biological processes (BPs). These processes were primarily related to nervous system regulation, response to lipopolysaccharide, signal transduction, hypoxia response, the gamma-aminobutyric acid (GABA) signaling pathway, and regulation of membrane potential (Figure 2A). Furthermore, the analysis revealed 36 distinct entries linked to cellular components (CCs), including key entities such as the cytoplasm, membrane receptors, and extracellular regions. Additionally, 62 entries were identified for molecular functions (MFs), encompassing critical activities such as ion channel binding, receptor activity, and heme binding, among others.

Subsequent enrichment analysis of KEGG pathways revealed their significant involvement in various critical signaling cascades (Figure 2B). These pathways primarily included the PI3K-AKT signaling pathway, the relaxin signaling pathway, the HIF-1 signaling pathway, the VEGF signaling pathway, the FoxO signaling pathway, the apelin signaling pathway, the calcium signaling pathway, the AGE-RAGE signaling pathway in diabetic complications, the chemokine signaling pathway, the metabolic pathway, the cGMP-PKG signaling pathway, and the JAK-STAT signaling pathway.

### 2.4. Construction of the Target–Disease Network

The intricate network illustrating the targets and pathways influenced by 3-MA in addressing DE conditions is visually represented in Figure 2C. In the network, pink nodes denote targets shared by 3-MA and DE, red nodes represent diseases, blue nodes represent drugs, and yellow and green nodes delineate the relevant signaling pathways identified through KEGG enrichment analysis. The “drug–disease–target–pathway” network diagram was constructed using Cytoscape software (version 3.10.0). Topological analysis revealed an average degree value of 6.9, with the top targets including AKT1, PTGS2, EGFR, HSP90AA 1, and GSK3β.

### 2.5. Predicting Active Compounds of 3-MA Through Molecular Docking

**Figure 3:**
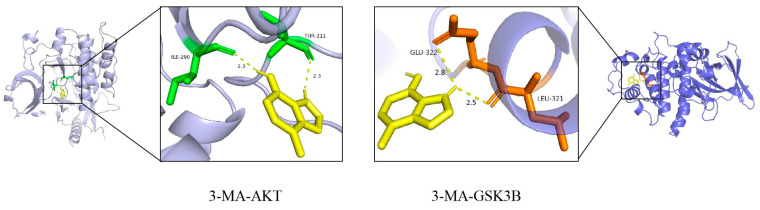
Visualization of molecular docking results of 3-MA with AKT and GSK3β proteins. The purple and blue structures represent AKT and GSK3β proteins, respectively; the yellow structure represents the 3-MA active compound; and the green and brown structures represent the binding sites between the two, respectively.

The molecular docking analysis of 3-MA with the core targets AKT1 and GSK3β revealed binding energies of −6.0 kcal/mol and −5.3 kcal/mol, respectively. These results demonstrate that 3-MA effectively binds to both AKT and GSK-3β proteins in their native states, with the binding conformation exhibiting significant stability. As illustrated in Figure 3, the molecular interactions are represented, with hydrogen bonds depicted as dashed lines, while distinct color coding was employed to differentiate between protein and ligand binding sites. Furthermore, the small molecule establishes hydrophobic interactions with key amino acid residues, including ILE-290, THR-211, GLU-322, and LEU-321. The formation of hydrogen bonds between the ligand and these amino acid residues enhances molecular activity. These findings collectively suggest that the synergistic effects of hydrogen bonding and hydrophobic interactions contribute to the strong binding affinity observed between 3-MA and the target proteins.

### 2.6. Effects of 3-MA on RBG and Body Weight

**Figure 4:**
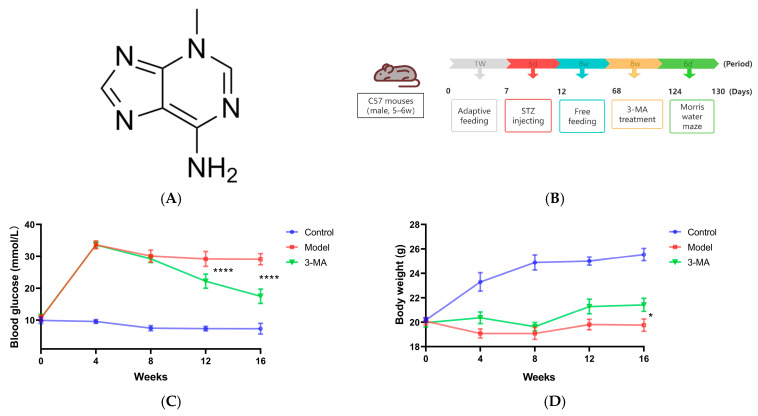
(**A**) The chemical structure of 3-MA. (**B**) Timeline diagram of animal model induction and 3-MA administration. (**C**) Random blood glucose. (**D**) Body weight. Data are shown as means ± SDs (*n* = 8 in each group). * *p* < 0.05 and **** *p* < 0.0001 compared to model.

The random blood glucose (RBG) levels in the DM group mice exhibited a progressive increase following streptozotocin (STZ) injection, consistently exceeding 16.7 mmol/L throughout the experimental period. Pharmacological intervention analysis revealed that compared with the DM group, the 3-MA treatment group showed significant reductions in RBG levels at both 12 and 16 weeks post-treatment (*** *p* < 0.001). Furthermore, a notable increase in body weight was observed in the 3-MA group at week 16 (* *p* < 0.05) (Figure 4C,D). Throughout the experimental duration, the DM group mice maintained a persistent hyperglycemic state and manifested characteristic diabetic symptoms, including pronounced polyuria, polydipsia, and polyphagia. These animals also displayed additional physiological alterations, notably a grayish-yellow fur coloration and reduced behavioral activity.

### 2.7. Effects of 3-MA on Learning and Memory Behavior

**Figure 5:**
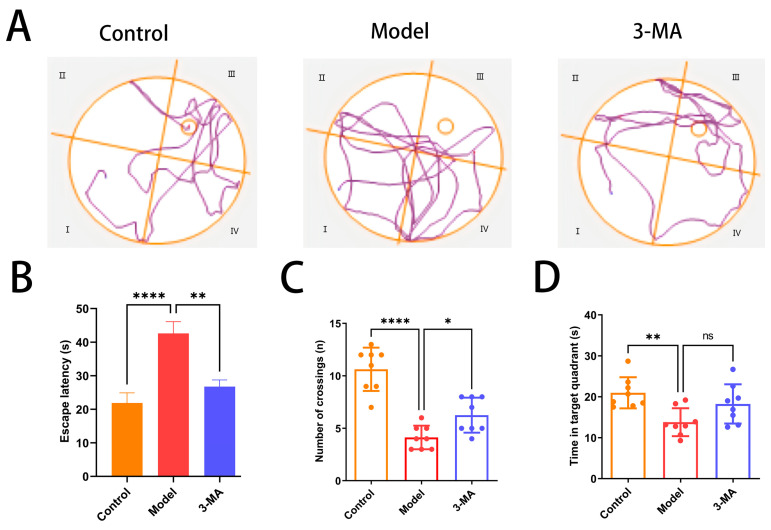
3-MA treatment ameliorates cognitive dysfunction in model mice. (**A**) Movement trajectories in Morris water maze test. (**B**) Escape latency. (**C**) Number of target quadrant crossings. (**D**) Target quadrant dwell time. Data are shown as means ± SDs (*n* = 8 in each group). * *p* < 0.05, ** *p* < 0.01, and **** *p* < 0.0001 compared to model. ns = no significance.

The Morris water maze test was conducted to evaluate spatial learning and memory capabilities in mice. During the localization navigation trials, significant differences were observed between the experimental groups. Compared to the control group, the model group demonstrated substantially prolonged path lengths to the original platform location and increased escape latency (**** *p* < 0.0001). Notably, 3-MA administration resulted in significant improvements, with both path length and escape latency being markedly reduced compared to the model group (** *p* < 0.01; Figure 5A,B). In the spatial exploration trials, the model group exhibited significantly impaired performance relative to the controls, as evidenced by reduced platform crossing frequency (**** *p* < 0.0001; Figure 5C) and decreased time spent in the target quadrant (** *p* < 0.01; Figure 5D). While 3-MA treatment significantly enhanced platform crossing frequency compared to the model group (* *p* < 0.05; Figure 5C), no statistically significant difference was observed in the time spent in the target quadrant between these groups (Figure 5D).

### 2.8. Effects of 3-MA on the Hippocampus Histopathology

**Figure 6:**
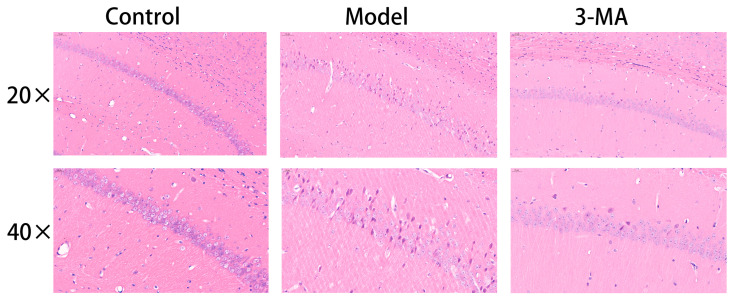
Effect of 3-MA on morphological changes in hippocampus CA1 region in model mice.

Histopathological examination revealed distinct morphological differences in hippocampal tissue architecture among the experimental groups. In the control group mice, the hippocampal structure maintained an intact cytoarchitecture, with pyramidal neurons in the CA1 region demonstrating a regular spatial organization; a uniform distribution; well-defined cellular contours; and compact, spherical nuclei. In contrast, the model group exhibited substantial hippocampal tissue damage, characterized by disorganized neuronal arrangements, significant cellular depletion, expanded interstitial spaces, indistinct cellular boundaries, and marked morphological abnormalities. Notably, 3-MA treatment group showed considerable neuroprotective effects, with partial restoration of hippocampal tissue integrity. Morphological analysis indicated improved neuronal organization, reduced cellular damage, and a relatively preserved cytoarchitecture compared to the model group (Figure 6).

### 2.9. Effect of 3-MA on the Expressions of APP and Tau Protein in Hippocampus

**Figure 7:**
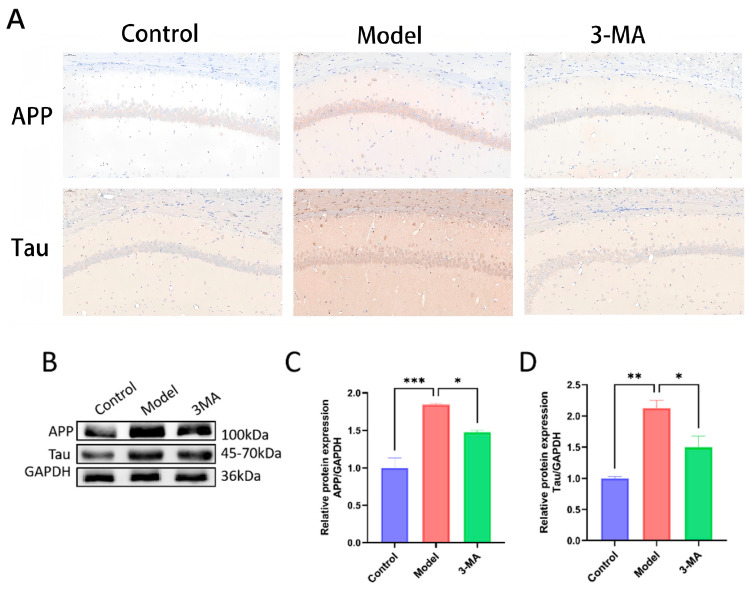
3-MA can reduce the levels of APP and Tau in the hippocampus. (**A**) Immunohistochemistry staining of APP and Tau in CA1 region (20×). (**B**) Western blot analysis of brain tissues after drug administration in each group of mice. (**C**) APP protein expression levels. (**D**) Tau protein expression levels. Data are shown as means ± SEMs (*n* = 3 in each group). * *p* < 0.05, ** *p* < 0.01, and *** *p* < 0.001 compared to model.

The APP and Tau positive neurons in the hippocampal neurons of the control group mice showed more uniform staining and regular contours. Compared with the control group, the model group showed an increase in the number of APP and Tau positive neurons in the hippocampal CA1 region, with darker staining and less clear contours. Compared with the model group, the 3-MA group showed a significant decrease in APP and Tau positive expression, with lighter staining (Figure 7A). Protein immunoblot results showed that the expression of APP and Tau proteins in the 3-MA group were significantly altered. Compared with the control group, the expression of APP protein in the model group mice was significantly increased (*** *p* < 0.001), and the expression of Tau protein was significantly increased (** *p* < 0.01). Compared with the model group, the expression of APP and Tau proteins in the 3-MA group mice was significantly decreased (* *p* < 0.05) (Figure 7C,D).

### 2.10. Western Blot Analysis of Brain Tissues

**Figure 8:**
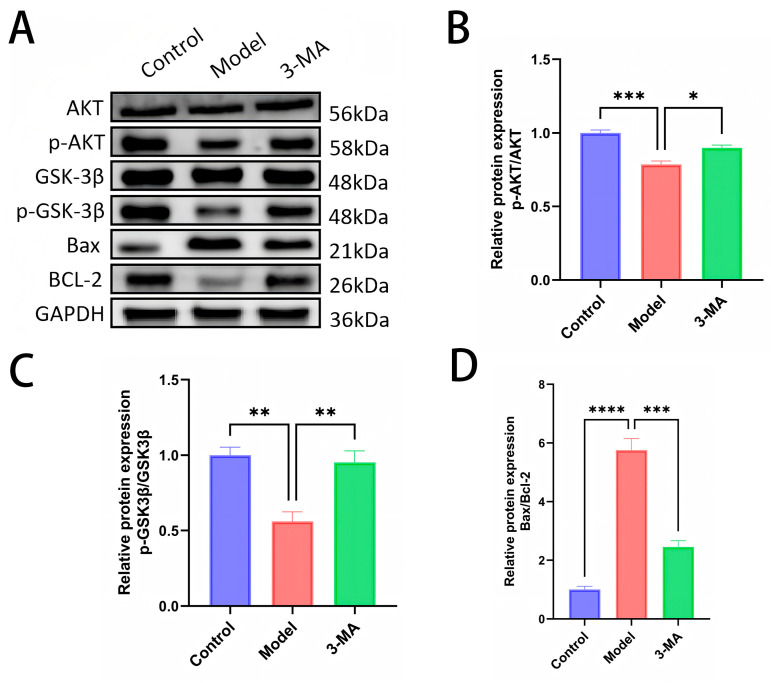
(**A**) Western blot analysis of brain tissues after drug administration in each group of mice. (**B**) p-AKT protein expression levels. (**C**) p-GSK3β protein expression levels. (**D**) Bax/BCL-2 protein ratios. Data are shown as means ± SEMs (*n* = 3 in each group). * *p* < 0.05, ** *p* < 0.01, *** *p* < 0.001, and **** *p* < 0.0001 compared to model.

Western blot analysis was performed to quantify the expression levels and phosphorylation status of AKT and GSK-3β proteins in brain tissues across the experimental groups (Figure 8B,C). Protein expression levels were determined by normalizing the grayscale values of phosphorylated proteins to their corresponding total protein levels. Comparative analysis revealed significant alterations in protein phosphorylation patterns. The model group exhibited markedly reduced levels of p-AKT and p-GSK-3β compared to the controls, with corresponding decreases in p-AKT/AKT (*** *p* < 0.001) and p-GSK-3β/GSK-3β (** *p* < 0.01) ratios. Notably, total protein expression of AKT and GSK-3β remained stable across all groups, showing no statistically significant differences. Following 3-MA treatment, significant modulation of the AKT/GSK-3β pathway was observed, characterized by increased p-AKT/AKT ratios (* *p* < 0.05) and significantly up-regulated p-GSK-3β/GSK-3β ratios (** *p* < 0.01) compared to the model group. These findings suggest that 3-MA may exert neuroprotective effects against diabetes-induced cognitive impairment through activation of the AKT/GSK-3β signaling pathway.

Western blot analysis was conducted to determine the expression profiles of the apoptosis-related proteins Bax and BCL-2 in brain tissues across the experimental groups (Figure 8D). Protein expression quantification was performed by calculating the grayscale ratio of Bax to BCL-2 immunoreactivity. Comparative analysis revealed significant dysregulation of apoptotic markers in the model group. Compared to the control animals, the model group mice exhibited markedly elevated Bax expression and significantly increased Bax/BCL-2 ratios (**** *p* < 0.0001). Notably, 3-MA treatment effectively attenuated these apoptotic changes, demonstrating significantly reduced Bax expression and decreased Bax/BCL-2 ratios compared to the model group (*** *p* < 0.001). These findings suggest that 3-MA exerts neuroprotective effects through the modulation of apoptotic pathways, specifically by regulating the expression of pro-apoptotic (Bax) and anti-apoptotic (BCL-2) factors, thereby ameliorating hippocampal neuronal damage in diabetic model mice.

## 3. Discussion

The global prevalence of diabetes has shown a consistent upward trend, paralleling changes in dietary patterns and lifestyle. Cognitive dysfunction has emerged as a significant complication in both type 1 and type 2 diabetes. Epidemiological studies indicate that diabetic patients face a 1.5- to 2-fold increased risk of developing cognitive decline, cognitive dysfunction, or dementia compared to non-diabetic individuals [27]. 3-MA, a well-characterized autophagy inhibitor frequently employed in autophagy research, is endogenously metabolized in biological systems. While primarily recognized for its autophagy-inhibitory properties, emerging evidence suggests that 3-MA exerts beneficial effects in non-autophagy-related conditions [28] and demonstrates therapeutic potential across various disease models [23,25,29]. The present study demonstrates that 3-MA administration effectively reduces blood glucose levels, suppresses apoptotic pathways, ameliorates diabetes-associated cognitive impairment, and enhances learning and memory functions in diabetic mice. Through comprehensive network pharmacological analysis and in vivo experimental validation, we have identified that 3-MA mediates its neuroprotective effects, at least in part, through activation of the AKT/GSK3β signaling pathway.

In this study, DM mice exhibited significant cognitive impairment, as evidenced by prolonged time to locate the target platform and increased path length during navigation training. Furthermore, in the spatial exploration test, these mice showed reduced time spent in the target quadrant and decreased frequency of crossing the target quadrant. Previous studies have demonstrated that 3-MA can ameliorate cognitive impairment associated with sepsis-associated encephalopathy (SAE) by inhibiting autophagy, both in the acute phase and during recovery [18]. Additionally, 3-MA has been reported to mitigate sevoflurane-induced cognitive dysfunction in neonatal mice, characterized by a reduced freezing time and fewer platform crossings [30]. In the context of Alzheimer’s disease (AD), 3-MA treatment has been shown to decrease escape latency in a mouse model with chronic cerebral hypoperfusion (CCH) [26]. Consistent with these findings, our study also observed that 3-MA administration significantly shortened the escape latency and swimming path length in DM mice, while increasing the number of platform crossings.

The pathogenesis of DE remains incompletely understood. Current evidence suggests that the accumulation of Aβ and hyperphosphorylated Tau protein in the brain constitutes a crucial pathological mechanism underlying DE development [31]. Postmortem studies have demonstrated elevated levels of Aβ and phosphorylated Tau in the brains of T2DM patients. These pathological features have been successfully replicated in animal models of T2DM [7]. Previous investigations have revealed that 3-MA can downregulate the expression of α-synuclein, amyloid precursor protein (APP), and phosphorylated Tau in the intestinal tissue of Parkinson’s disease dementia (PDD) mice, potentially alleviating PDD symptoms through the brain–gut axis pathway [32]. In the present study, we observed significant downregulation of APP and Tau protein expression in hippocampal tissues of 3-MA-treated mice compared to the model group. However, these findings appear contradictory to some previous reports suggesting that 3-MA might enhance APP expression and aggregation, consequently promoting Aβ production. Furthermore, 3-MA has been proposed to indirectly influence Tau phosphorylation and aggregation through modulation of autophagy pathways [33]. The discrepancy in research outcomes may be attributed to variations in autophagy homeostasis or potential interactions between autophagy and non-autophagy pathways, though the precise mechanisms warrant further investigation. Our findings collectively suggest that 3-MA exerts therapeutic effects on DE through multiple mechanisms: (1) reducing escape latency in diabetic mice, (2) ameliorating cognitive deficits, and (3) decreasing APP and Tau aggregation in brain tissues.

The accumulation of Aβ and p-Tau proteins has been demonstrated to induce neuronal apoptosis, leading to significant impairments in memory and learning functions [34]. 3-MA exhibits considerable potential in apoptosis regulation. Comparative studies have revealed that 3-MA significantly reduces oxidative damage and cellular apoptosis when compared with the autophagy inducer rapamycin [35]. Furthermore, pretreatment with 3-MA has been shown to effectively decrease apoptosis levels and mitigate associated morphological alterations. Notably, 3-MA has demonstrated protective effects against pulmonary microvascular endothelial cell damage through its anti-apoptotic properties [24]. In the current study, we observed significant upregulation of BCL-2 expression and a corresponding decrease in the Bax/BCL-2 ratio in 3-MA-treated diabetic mice (Figure 8D), which aligns with previously established findings. Additionally, 3-MA treatment effectively restored the morphological integrity of hippocampal tissues and markedly ameliorated neuronal damage. These findings collectively suggest that 3-MA ameliorates cognitive impairment in diabetic mice through its anti-apoptotic mechanisms.

Accumulating evidence has demonstrated that the AKT/GSK3β signaling pathway plays a crucial role in regulating various physiological processes, including glucose and lipid metabolism, central nervous system (CNS) cell apoptosis, neurogenesis, synaptic plasticity, and oxidative stress. Dysregulation of this pathway has been closely associated with the pathogenesis and progression of DE [36,37,38]. AKT1, a critical growth and survival factor, mediates adaptive β-cell responses and plays compensatory roles in neuronal insulin signaling, glucose uptake, and insulin resistance regulation [39]. GSK3β, a downstream target of the PI3K/AKT pathway, is negatively regulated by AKT1. This kinase is abundantly expressed throughout the CNS, particularly in the hippocampus, where it significantly contributes to memory formation and cognitive functions [40]. The activation of GSK3β has been shown to promote Aβ production and aggregation, leading to excessive Tau protein phosphorylation and subsequent neurofibrillary tangle formation [41], ultimately resulting in neuronal damage and cognitive deficits [42]. Conversely, activation of the AKT/GSK3β pathway has demonstrated neuroprotective and repair capabilities [43]. In the current study, we investigated the effects of 3-MA on key components of the AKT/GSK3β pathway in the hippocampus of STZ-induced diabetic mice. As illustrated in Figure 8B,C, compared to the control group, diabetic mice exhibited significantly reduced phosphorylation levels of AKT and GSK3β in hippocampal tissues, while total protein levels remained unchanged. Notably, 3-MA treatment effectively restored the phosphorylation levels of both AKT and GSK3β. These findings suggest that 3-MA ameliorates cognitive impairment in diabetic mice through activation of the AKT/GSK3β signaling pathway.

Extensive research has demonstrated that hyperglycemia-induced DE significantly impairs cognitive function and elevates the risk of neurodegenerative disorders in both diabetic patients and animal models [44]. Experimental evidence indicates that chronic hyperglycemia facilitates Aβ aggregation in cerebral tissues, compromises neuronal structural integrity, and ultimately leads to progressive neurodegeneration [45]. In the present study, 3-MA administration effectively attenuated hyperglycemia in diabetic mice, consequently reducing Aβ deposition and ameliorating cognitive deficits. These findings suggest that the neuroprotective efficacy of 3-MA may be partially mediated through its glucose-lowering properties.

Autophagy, a fundamental cellular protective mechanism, requires precise regulation as both excessive and insufficient autophagic activity can detrimentally affect cellular homeostasis and organismal health [46]. This lysosomal degradation pathway plays a critical role in eliminating cytotoxic protein aggregates in the brain, exerting neuroprotective effects in various neurodegenerative conditions [47]. Particularly in the hippocampus, dysregulated neuronal autophagy has been associated with cellular damage and cognitive dysfunction [48]. Guo et al. [49] demonstrated that intracerebroventricular administration of 3-MA effectively inhibits resveratrol-induced autophagy activation, consequently delaying neurological functional recovery. As a well-characterized PI3K inhibitor, 3-MA modulates autophagic activity by specifically targeting the initiation phase of autophagy. The therapeutic potential of 3-MA in neuroprotection and cognitive enhancement depends on the autophagic status: in conditions of excessive autophagy leading to neuronal damage, 3-MA exerts beneficial effects through autophagy inhibition. Conversely, in cases of autophagic insufficiency, 3-MA administration may potentially exacerbate pathological conditions.

While this study provides preliminary insights into 3-MA’s potential mechanisms, several limitations should be noted. First, although our molecular docking analysis employed multiple scoring functions (e.g., AutoDock Vina), further validation through molecular dynamics simulations and binding free energy calculations (MM-PBSA/MM-GBSA) would strengthen the assessment of binding stability and affinity under physiological conditions. Second, while our computational predictions are supported by phenotypic observations, direct experimental validation is needed to confirm target engagement, such as enzyme inhibition or binding assays. Additionally, although we did not measure autophagy-related indicators, the potential role of 3-MA-mediated autophagy regulation in diabetic encephalopathy warrants further investigation to elucidate its precise contribution under hyperglycemic conditions. These limitations highlight key areas for future research to more definitively establish 3-MA’s mechanism of action and therapeutic potential.

## 4. Materials and Methods

### 4.1. Materials and Reagents

Methyladenine (M2296) was purchased from Abmole Co., Ltd. (Shanghai, China). Streptozotocin (STZ, V900890) and PVDF membrane (PR05505) were obtained from Sigma Co., Ltd. (Beijing, China). PBS buffer (powder, BL601A) was acquired from BioSharp (Hefei, China). The following antibodies and reagents were purchased from Proteintech Group, Inc. (Wuhan, China): AKT (10176-2-AP, 1:5000), phosphorylated AKT (p-AKT, 28731-1-AP, 1:3000), GSK-3β (22104-1-AP, 1:4000), phosphorylated GSK-3β (p-GSK-3β, 14850-1-AP, 1:2000), Bax (50599-2-Ig, 1:5000), BCL-2 (26593-1-AP, 1:1000), APP (25524-1-AP, 1:1000), Tau (10274-1-AP, 1:7000), and HRP-labeled goat anti-rabbit IgG (RGAR001, 1:5000). GAPDH antibody (bs-6951R) was sourced from Boosen (Beijing, China). RIPA lysis buffer (G2002) and 5× protein loading buffer (G2075) were procured from Sevier (Wuhan, China). The BCA protein quantification kit (KR0008) and ECL hypersensitive chemiluminescence reagent (KR0006) were obtained from Kerui Biological Co., Ltd. (Wuhan, China). Titanium dioxide (AEROXIDE P26) was purchased from Rhine Chemical Co., Ltd. (Shanghai, China).

### 4.2. 3-MA Target Prediction

The Simplified Molecular Input Line Entry System (SMILES) identifiers for 3-MA were retrieved from the PubChem database (https://pubchem.ncbi.nlm.nih.gov/, accessed on 24 July 2024) [50]. These identifiers were subsequently input into the Swiss Target Prediction database (http://www.swisstargetprediction.ch/, accessed on 24 July 2024) [51], the PharmMapper database (https://www.lilab-ecust.cn/pharmmapper/, accessed on 25 July 2024) [52], and the SEA database (https://sea.bkslab.org/, accessed on 25 July 2024) [53]. Potential targets of 3-MA were predicted by selecting the species “Homo sapiens”, and the results were downloaded in CSV format for further analysis.

### 4.3. Search for DE-Related Genes

The keyword “diabetic encephalopathy” was used to search for potential disease-associated targets within the GeneCards (https://www.genecards.org/, accessed on 26 July 2024) [54], OMIM (https://www.omim.org/, accessed on 26 July 2024) [55], and DisGeNET databases (https://www.disgenet.org/, accessed on 26 July 2024) [56]. Based on the relevance score, which reflects the strength of the association between potential targets and the disease, the identified targets were screened by ranking the scores from highest to lowest. A threshold of three times the median relevance score was applied, and the results were subsequently merged and deduplicated to obtain DE-related target genes.

### 4.4. Construction of Protein–Protein Interaction (PPI) Network and SeIection of Key Targets

An interactive Venn diagram was generated by inputting the predicted targets of 3-MA and the DE-related target genes into the VENNY 2.1 online tool (http://www.bioinformatics.com.cn/, accessed on 27 July 2024) [57]. The overlapping region of the diagram represents the intersection of 3-MA and DE targets. The intersecting target genes were subsequently uploaded to the STRING 12.0 database (https://string-db.org/, accessed on 27 July 2024) [58], with the species set to Homo sapiens. A high-resolution protein–protein interaction (PPI) network was constructed and exported in TSV format. The TSV file was then imported into Cytoscape 3.10.0 software for network visualization. Core targets were identified using topological parameters, including degree centrality (DC), betweenness centrality (BC), and closeness centrality (CC), calculated by the “CytoNCA” plugin in Cytoscape.

### 4.5. Cytoscape Gene OntoIogy (GO) and Kyoto EncycIopedia of Gene and Genomes (KEGG) Enrichment AnaIysis

The intersecting targets of 3-MA and DE were uploaded to the online bioinformatics analysis platform (http://www.bioinformatics.com.cn/, accessed on 1 August 2024) [59] for Gene Ontology (GO) and Kyoto Encyclopedia of Genes and Genomes (KEGG) enrichment analyses. The top 20 significant terms were selected based on their relevance to molecular function (MF), biological process (BP), and cellular component (CC) categories. Similarly, 12 primary signaling pathways were identified from the 67 enriched pathways for further analysis.

### 4.6. Constructing the “Drug–Disease–Target–Pathway” Network

The key pathways, intersecting targets, and 3-MA- and DE-related data were organized into an Excel sheet to generate a Network and Type tool file. This file was subsequently imported into Cytoscape 3.10.0 software [60] to construct a “3-MA–disease–target–pathway” network diagram. The construction of the network was referenced from Du’s study [61]. In this network, nodes represent target genes associated with the bioactive components of 3-MA and DE, while edges illustrate the interactions between 3-MA, its targets, pathways, and the disease.

### 4.7. MoIecuIar Docking Verification

The molecular structure of 3-MA in mol2 format was obtained from the PubChem database (https://pubchem.ncbi.nlm.nih.gov/, accessed on 3 August 2024). The three-dimensional (3D) structures of the core targets (AKT and GSK3β) were retrieved from the RCSB Protein Data Bank (https://www.rcsb.org/, accessed on 3 August 2024) [62]. The selection criteria included experimentally validated, X-ray crystallography-resolved protein structures from Homo sapiens with a resolution of less than 3.0 Å. The protein structures were processed by removing water molecules and ligands, followed by hydrogenation, and saved in “pdbqt” format using AutoDock Tools 1.5.7 software [63]. Similarly, the mol2 structure of 3-MA was converted into the “pdbqt” format. Molecular docking was performed using AutoDock Tools 1.5.7, and the results were visualized using PyMOL 3.0 software [64].

### 4.8. Animals

Twenty-four specific pathogen-free (SPF)-grade male C57BL/6J mice, aged 5–6 weeks and weighing 18–22 g, were obtained from the Animal Experiment Center of Three Gorges University (license number: SCXK(E)-2022-0012). The mice were housed under controlled environmental conditions, including a temperature of 18–22 °C, a relative humidity of 40–60%, and a 12 h light/dark cycle. They were provided with ad libitum access to food and water. All experimental procedures were approved by the Ethics Committee of Yangtze University (approval number: YZLL2025-001).

### 4.9. Establishment of DE Model and Treatment of 3-MA

After one week of acclimatization, eight mice were randomly assigned to the normal control group (Control). The remaining mice were intraperitoneally administered 1% streptozotocin (STZ) solution (55 mg/kg/day) [65] for five consecutive days to establish the diabetes mellitus (DM) model, and the control group mice received an equivalent volume of sodium citrate buffer. On the third day after the final STZ injection, mice with random blood glucose (RBG) levels exceeding 16.7 mmol/L were included in the study. The successfully modeled mice were then divided into two groups (*n* = 8 each): the DM model group (Model) and the 3-methyladenine treatment group (3-MA group). Eight weeks after DM model induction, the 3-MA group was administered 3-MA via oral gavage at a dose of 10 mg/kg/day for eight weeks. The control and model groups received an equal volume of sterile phosphate-buffered saline (PBS) solution.

### 4.10. Random Blood Glucose and Body Weight Measurement

Body weight and blood glucose levels were monitored every four weeks. Blood glucose measurements were taken between 7:00 P.M. and 9:00 P.M. using an Accu-Chek Performa glucometer (Roche, Mannheim, Germany). Approximately 3 μL of blood was collected from conscious mice via the tail vein for each measurement.

### 4.11. Morris Water Maze

The effects of 3-MA on spatial learning and memory abilities in diabetic mice were evaluated using the Morris water maze test [66], which included a localization navigation test and a spatial exploration test. The water maze apparatus consisted of a circular pool (120 cm in diameter and 40 cm in height), a hidden platform (10 cm in diameter), and an overhead camera tracking system. The pool was divided into four equal quadrants, each marked with distinct visual cues on the walls. The water temperature was maintained at 22 ± 2 °C. Prior to each experiment, mice were acclimatized to the testing room for 30 min to ensure adaptation to the experimental environment. All behavioral data were recorded and analyzed using the Any-Maze 6.3 software tracking system.

Day 1 was the acclimatization period. The platform was placed in one of the quadrants, positioned 1.0 cm above the water surface. Mice that successfully located and climbed onto the platform were considered to have normal locomotion and vision, confirming their eligibility for subsequent experiments. Days 2–5 were designated for the localization navigation test, which lasted for 4 days. Titanium dioxide, a non-toxic pigment, was added to the water to render it opaque. The platform was placed in the target quadrant (Quadrant III) and submerged 1.0 cm below the water surface. Each mouse underwent four training sessions per day. During each session, the mice were placed into the water at the midpoint of different quadrants, facing the pool wall. The time taken for the mice to locate the platform through directional recognition was recorded as the escape latency. If a mouse failed to find the platform within 60 s, it was gently guided to the platform and allowed to remain there for 15 s to familiarize itself with the location. In such cases, the escape latency was recorded as 60 s. Day 6 was designated for the spatial exploration test. On the final day, the platform was removed. Each mouse was placed into the water at the farthest point from the original platform location (Quadrant I) and allowed to swim freely for 60 s. The number of times the mice crossed the target quadrant and the duration spent in the target quadrant were recorded as measures of spatial exploration ability.

### 4.12. H/E Staining Analysis

Hippocampal tissues from each group of mice were fixed in 4% paraformaldehyde solution. The tissues were then routinely processed through a series of steps, including dehydration in a graded alcohol series (50%, 60%, 70%, 80%, 90%, and 95%, each for 5 min), clearing in xylene, paraffin embedding, and continuous sectioning at a thickness of 4 μm. The sections were dewaxed, stained with hematoxylin and eosin (H&E), and mounted with neutral gum [67]. The morphological features of hippocampal neurons were examined under a light microscope.

### 4.13. IHC Analysis

Paraffin-embedded sections stored at room temperature were dewaxed in xylene and rehydrated through a graded ethanol series. Antigen retrieval was performed by incubating the brain sections in Tris-EDTA buffer at 100 °C for 20 min. The sections were then permeabilized with 0.3% Triton X-100 and blocked with 10% normal goat serum for 1 h at room temperature to prevent non-specific binding [67]. Subsequently, the sections were incubated overnight at 4 °C with primary antibodies against APP and Tau (diluted 1:200). After washing, the sections were incubated with secondary antibodies for 1 h at room temperature, followed by color development using 3,3′-diaminobenzidine (DAB) and counterstaining with hematoxylin. Images were captured using a Nikon E100 microscope (Tokyo, Japan).

### 4.14. Western Blot Analysis

Brain tissues were homogenized in RIPA lysis buffer supplemented with protease and phosphatase inhibitors, followed by centrifugation at 12,000 rpm for 30 min at 4 °C. The supernatant was collected, and the total protein concentration was quantified using the bicinchoninic acid (BCA) assay. Protein samples were separated by sodium dodecyl sulfate–polyacrylamide gel electrophoresis (SDS-PAGE) and subsequently transferred onto a polyvinylidene fluoride (PVDF) membrane. The membrane was blocked with 5% skimmed milk at room temperature for 2 h and then incubated overnight at 4 °C with primary antibodies specific to the target proteins [68]. After washing three times with Tris-buffered saline containing Tween 20 (TBST) for 10 min each, the membrane was incubated with horseradish peroxidase (HRP)-conjugated secondary antibodies at room temperature for 1 h. Following another three washes with TBST, protein bands were visualized using an enhanced chemiluminescence (ECL) ultrasensitive detection kit and imaged using a chemiluminescence imaging system. The resulting data were analyzed using ImageJ 1.4.3 software.

### 4.15. Statistical Analysis

All data were statistically analyzed using SPSS 17.0 software. All data were expressed as means ± SEMs/SDs. Comparisons between multiple groups were performed using one-way ANOVA, and comparisons between two groups were performed using the LSD *t*-test. *p* < 0.05 was considered statistically significant.

## 5. Conclusions

Accumulating evidence underscores the significant impact of lifestyle factors, particularly dietary patterns, on promoting healthy aging and preserving cognitive function. Numerous studies have substantiated that lifestyle modifications, with emphasis on dietary interventions, effectively mitigate age-related cognitive decline [69]. Our findings demonstrate that 3-MA modulates the phosphorylation of Akt and GSK-3β, ameliorates insulin resistance, regulates blood glucose homeostasis, and alleviates cognitive dysfunction in diabetic murine models. These results suggest that dietary supplementation with 3-MA may offer both preventive and therapeutic potential for DE in clinical settings. In conclusion, through the integrative approach combining network pharmacology analysis and in vivo experimentation, we have elucidated potential molecular targets and mechanisms underlying 3-MA’s intervention in DE. Our findings indicate that 3-MA exerts neuroprotective effects and enhances cognitive performance. Furthermore, molecular docking studies have provided insights into the structural basis of 3-MA’s therapeutic activity against DE. These findings establish a foundation for future investigations into 3-MA-based therapeutic strategies for DE, offering valuable directions and conceptual frameworks for subsequent research.

## Data Availability

Data are contained within the article.
